# Supplementary material for: Investigation of Functional Connectivity Differences between Voluntary Respirations via Mouth and Nose Using Resting State fMRI
Source: Brain Sci. 2020 Oct 3;10(10):704. doi: 10.3390/brainsci10100704 (PMC7599777; doi:10.3390/brainsci10100704)
Supplement: Supplementary file 1 [file brainsci-10-00704-s001.pdf]

Supplementary Materials: Investigation of Functional Connectivity Differences between Voluntary Respirations via Mouth and Nose using Resting State fMRI

Ju-Yeon Jung [1], Chan-A Park [2], Yeong-Bae Lee [3,4], Chang-Ki Kang [4,5,6,*]

Table S1. 164 ROIs used as functional connectivity analysis.

ROI			
1	atlas.FP r (Frontal Pole Right)	83	atlas.PP l (Planum Polare Left)
2	atlas.FP l (Frontal Pole Left)	84	atlas.HG r (Heschl's Gyrus Right)
3	atlas.IC r (Insular Cortex Right)	85	atlas.HG l (Heschl's Gyrus Left)
4	atlas.IC l (Insular Cortex Left)	86	atlas.PT r (Planum Temporale Right)
5	atlas.SFG r (Superior Frontal Gyrus Right)	87	atlas.PT l (Planum Temporale Left)
6	atlas.SFG l (Superior Frontal Gyrus Left)	88	atlas.SCC r (Supracalcarine Cortex Right)
7	atlas.MidFG r (Middle Frontal Gyrus Right)	89	atlas.SCC l (Supracalcarine Cortex Left)
8	atlas.MidFG l (Middle Frontal Gyrus Left)	90	atlas.OP r (Occipital Pole Right)
9	atlas.IFG tri r (Inferior Frontal Gyrus, pars triangularis Right)	91	atlas.OP l (Occipital Pole Left)
10	atlas.IFG tri l (Inferior Frontal Gyrus, pars triangularis Left)	92	atlas.Thalamus r
11	atlas.IFG oper r (Inferior Frontal Gyrus, pars opercularis Right)	93	atlas.Thalamus l
12	atlas.IFG oper l (Inferior Frontal Gyrus, pars opercularis Left)	94	atlas.Caudate r
13	atlas.PreCG r (Precentral Gyrus Right)	95	atlas.Caudate l
14	atlas.PreCG l (Precentral Gyrus Left)	96	atlas.Putamen r
15	atlas.TP r (Temporal Pole Right)	97	atlas.Putamen l
16	atlas.TP l (Temporal Pole Left)	98	atlas.Pallidum r
17	atlas.aSTG r (Superior Temporal Gyrus, anterior division Right)	99	atlas.Pallidum l
18	atlas.aSTG l (Superior Temporal Gyrus, anterior division Left)	100	atlas.Hippocampus r
19	atlas.pSTG r (Superior Temporal Gyrus, posterior division Right)	101	atlas.Hippocampus l
20	atlas.pSTG l (Superior Temporal Gyrus, posterior division Left)	102	atlas.Amygdala r
21	atlas.aMTG r (Middle Temporal Gyrus, anterior division Right)	103	atlas.Amygdala l
22	atlas.aMTG l (Middle Temporal Gyrus, anterior division Left)	104	atlas.Accumbens r
23	atlas.pMTG r (Middle Temporal Gyrus, posterior division Right)	105	atlas.Accumbens l
24	atlas.pMTG l (Middle Temporal Gyrus, posterior division Left)	106	atlas.Brain-Stem
25	atlas.toMTG r (Middle Temporal Gyrus, temporooccipital part Right)	107	atlas.Cereb1 l (Cerebelum Crus1 Left)
26	atlas.toMTG l (Middle Temporal Gyrus, temporooccipital part Left)	108	atlas.Cereb1 r (Cerebelum Crus1 Right)
27	atlas.aITG r (Inferior Temporal Gyrus, anterior division Right)	109	atlas.Cereb2 l (Cerebelum Crus2 Left)
28	atlas.aITG l (Inferior Temporal Gyrus, anterior division Left)	110	atlas.Cereb2 r (Cerebelum Crus2 Right)

29	atlas.pITG r (Inferior Temporal Gyrus, posterior division Right)	111	atlas.Cereb3 l (Cerebelum 3 Left)
30	atlas.pITG l (Inferior Temporal Gyrus, posterior division Left)	112	atlas.Cereb3 r (Cerebelum 3 Right)
31	atlas.toITG r (Inferior Temporal Gyrus, temporooccipital part Right)	113	atlas.Cereb45 l (Cerebelum 4 5 Left)
32	atlas.toITG l (Inferior Temporal Gyrus, temporooccipital part Left)	114	atlas.Cereb45 r (Cerebelum 4 5 Right)
33	atlas.PostCG r (Postcentral Gyrus Right)	115	atlas.Cereb6 l (Cerebelum 6 Left)
34	atlas.PostCG l (Postcentral Gyrus Left)	116	atlas.Cereb6 r (Cerebelum 6 Right)
35	atlas.SPL r (Superior Parietal Lobule Right)	117	atlas.Cereb7 l (Cerebelum 7b Left)
36	atlas.SPL l (Superior Parietal Lobule Left)	118	atlas.Cereb7 r (Cerebelum 7b Right)
37	atlas.aSMG r (Supramarginal Gyrus, anterior division Right)	119	atlas.Cereb8 l (Cerebelum 8 Left)
38	atlas.aSMG l (Supramarginal Gyrus, anterior division Left)	120	atlas.Cereb8 r (Cerebelum 8 Right)
39	atlas.pSMG r (Supramarginal Gyrus, posterior division Right)	121	atlas.Cereb9 l (Cerebelum 9 Left)
40	atlas.pSMG l (Supramarginal Gyrus, posterior division Left)	122	atlas.Cereb9 r (Cerebelum 9 Right)
41	atlas.AG r (Angular Gyrus Right)	123	atlas.Cereb10 l (Cerebelum 10 Left)
42	atlas.AG l (Angular Gyrus Left)	124	atlas.Cereb10 r (Cerebelum 10 Right)
43	atlas.sLOC r (Lateral Occipital Cortex, superior division Right)	125	atlas.Ver12 (Vermis 1 2)
44	atlas.sLOC l (Lateral Occipital Cortex, superior division Left)	126	atlas.Ver3 (Vermis 3)
45	atlas.iLOC r (Lateral Occipital Cortex, inferior division Right)	127	atlas.Ver45 (Vermis 4 5)
46	atlas.iLOC l (Lateral Occipital Cortex, inferior division Left)	128	atlas.Ver6 (Vermis 6)
47	atlas.ICC r (Intracalcarine Cortex Right)	129	atlas.Ver7 (Vermis 7)
48	atlas.ICC l (Intracalcarine Cortex Left)	130	atlas.Ver8 (Vermis 8)
49	atlas.MedFC (Frontal Medial Cortex)	131	atlas.Ver9 (Vermis 9)
50	atlas.SMA r (Juxtapositional Lobule Cortex -formerly Supplementary Motor Cortex- Right)	132	atlas.Ver10 (Vermis 10)
51	atlas.SMA L(Juxtapositional Lobule Cortex -formerly Supplementary Motor Cortex- Left)	133	networks.DefaultMode.MPFC (1,55,-3)
52	atlas.SubCalC (Subcallosal Cortex)	134	networks.DefaultMode.LP (L) (-39,-77,33)
53	atlas.PaCiG r (Paracingulate Gyrus Right)	135	networks.DefaultMode.LP (R) (47,-67,29)
54	atlas.PaCiG l (Paracingulate Gyrus Left)	136	networks.DefaultMode.PCC (1,-61,38)
55	atlas.AC (Cingulate Gyrus, anterior division)	137	networks.SensoriMotor.Lateral (L) (-55,-12,29)
56	atlas.PC (Cingulate Gyrus, posterior division)	138	networks.SensoriMotor.Lateral (R) (56,-10,29)
57	atlas.Precuneous (Precuneous Cortex)	139	networks.SensoriMotor.Superior (0,-31,67)
58	atlas.Cuneal r (Cuneal Cortex Right)	140	networks.Visual.Medial (2,-79,12)
59	atlas.Cuneal l (Cuneal Cortex Left)	141	networks.Visual.Occipital (0,-93,-4)
60	atlas.FOrb r (Frontal Orbital Cortex Right)	142	networks.Visual.Lateral (L) (-37,-79,10)
61	atlas.FOrb l (Frontal Orbital Cortex Left)	143	networks.Visual.Lateral (R) (38,-72,13)
62	atlas.aPaHC r (Parahippocampal Gyrus, anterior division Right)	144	networks.Salience.ACC (0,22,35)
63	atlas.aPaHC l (Parahippocampal Gyrus, anterior division Left)	145	networks.Salience.AInsula (L) (-44,13,1)
64	atlas.pPaHC r (Parahippocampal Gyrus, posterior division Right)	146	networks.Salience.AInsula (R) (47,14,0)
65	atlas.pPaHC l (Parahippocampal Gyrus, posterior division Left)	147	networks.Salience.RPFC (L) (-32,45,27)
66	atlas.LG r (Lingual Gyrus Right)	148	networks.Salience.RPFC (R) (32,46,27)

67	atlas.LG l (Lingual Gyrus Left)	149	networks.Salience.SMG (L) (-60,-39,31)
68	atlas.aTFusC r (Temporal Fusiform Cortex, anterior division Right)	150	networks.Salience.SMG (R) (62,-35,32)
69	atlas.aTFusC l (Temporal Fusiform Cortex, anterior division Left)	151	networks.DorsalAttention.FEF (L) (-27,-9,64)
70	atlas.pTFusC r (Temporal Fusiform Cortex, posterior division Right)	152	networks.DorsalAttention.FEF (R) (30,-6,64)
71	atlas.pTFusC l (Temporal Fusiform Cortex, posterior division Left)	153	networks.DorsalAttention.IPS (L) (-39,-43,52)
72	atlas.TOFusC r (Temporal Occipital Fusiform Cortex Right)	154	networks.DorsalAttention.IPS (R) (39,-42,54)
73	atlas.TOFusC l (Temporal Occipital Fusiform Cortex Left)	155	networks.FrontoParietal.LPFC (L) (-43,33,28)
74	atlas.OFusG r (Occipital Fusiform Gyrus Right)	156	networks.FrontoParietal.PPC (L) (-46,-58,49)
75	atlas.OFusG l (Occipital Fusiform Gyrus Left)	157	networks.FrontoParietal.LPFC (R) (41,38,30)
76	atlas.FO r (Frontal Operculum Cortex Right)	158	networks.FrontoParietal.PPC (R) (52,-52,45)
77	atlas.FO l (Frontal Operculum Cortex Left)	159	networks.Language.IFG (L) (-51,26,2)
78	atlas.CO r (Central Opercular Cortex Right)	160	networks.Language.IFG (R) (54,28,1)
79	atlas.CO l (Central Opercular Cortex Left)	161	networks.Language.pSTG (L) (-57,-47,15)
80	atlas.PO r (Parietal Operculum Cortex Right)	162	networks.Language.pSTG (R) (59,-42,13)
81	atlas.PO l (Parietal Operculum Cortex Left)	163	networks.Cerebellar.Anterior (0,-63,-30)
82	atlas.PP r (Planum Polare Right)	164	networks.Cerebellar.Posterior (0,-79,-32)

Table S2. Statistical result for mean and variance of GSs of mouth and nose breathings.

	**Statistics (*T*)**	***P* value**
GS mean	-0.178	0.861
GS variance	-0.605	0.552

Abbreviations: GS, global signal.

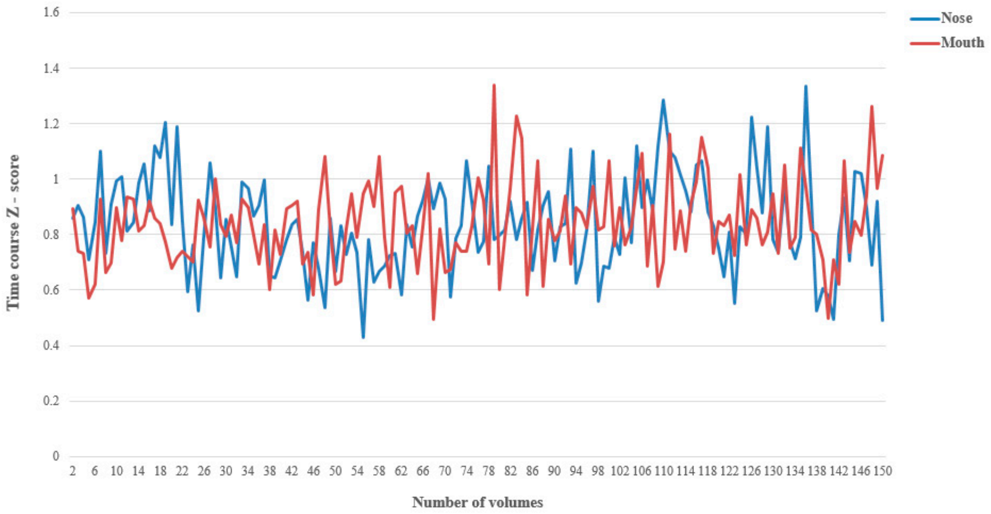

Figure S1. Mean time-course signals presented with Z – scores of GS. Blue legend represented "Nose" breathing condition and red legend represented "Mouth" breathing condition.
